# Community knowledge and attitude towards mental illness and its associated factors in Dodoma urban central Tanzania: A cross-sectional study

**DOI:** 10.1371/journal.pmen.0000267

**Published:** 2025-03-17

**Authors:** Erick Donard Oguma, Elihuruma Eliufoo Stephano, Fabiola Vincent Moshi, Osward Sevin Lyimo, Stephen M. Kibusi

**Affiliations:** 1 Department of Clinical Nursing, School of Nursing and Public Health, The University of Dodoma, Dodoma, Tanzania; 2 Department of Adolescent and Youth Mental Health, Tanzania Youth Bright Future Organization, Dodoma, Tanzania,; 3 Department of Nursing Management and Education, School of Nursing and Public Health, The University of Dodoma, Dodoma, Tanzania; 4 Department of Public Health and Community Nursing, The University of Dodoma, Dodoma, Tanzania; PLOS: Public Library of Science, UNITED KINGDOM OF GREAT BRITAIN AND NORTHERN IRELAND

## Abstract

Access to mental health services and care is hindered by stigmatizing attitude and lack of community knowledge about mental illness. The study aimed to assess the community knowledge and attitude towards mental illness and its associated factors in Dodoma urban, central Tanzania. A community-based analytical cross-sectional study was conducted among 204 participants in Dodoma urban central Tanzania, from July to September 2021. A structured questionnaire adapted from the previous studies was used to collect data from the study participants. A multistage sampling technique was used to select study participants. The factors associated with community knowledge and attitude toward mental illness were established using bivariable and multivariable binary logistic regression models. Majority of respondents have adequate knowledge (67.6%) and positive attitude (55.4%) toward mental illness. Unwilling to visit (61.3%), marry (80.4%), and share a room (52%), were the common identified community stigmatizing attitude toward people with mental illness. The multivariable binary logistic regression model revealed that, age [aOR = 9.94: 95% CI; 1.04–94.97: p = 0.046], ethnicity [aOR = 4.45: 95% CI; 1.20–16.53: p = 0.026], and occupation [aOR = 0.19: 95% CI; 0.04–0.85: p = 0.029] were the social demographic factors associated with community attitude toward mental illness. While, sex [aOR = 5.85: 95% CI; 1.77–19.34: p = 0.004], religion [aOR = 0.36: 95% CI; 0.14–0.93: p = 0.034], marital status [aOR = 0.16: 95% CI; 0.03–0.9: p = 0.039], and occupation [aOR = 18.69: 95% CI; 2.10–166.79: p = 0.009] were the social demographic factors associated with community knowledge regarding mental illness. The study revealed that more than half of respondents displayed a common stigmatizing attitude of not being willing to share a room, marry, or visit a mentally ill person, despite the participants’ adequate knowledge and favorable attitude. Using strategies like the provision of mental health education, forming community-based mental health organizations, and launching public mental health campaigns will help to improve community mental health awareness and reduce negative attitudes.

## Introduction

Mental disorders have remained a significant global challenge for many years [1], it contributes to social, occupational, and functional impairment [2]. It affects an individual judgments, cognition, perception, and behavior [3]. Globally, one out of every eight people which is approximately 970 million people, suffers from a mental disorder [4]. Anxiety and depressive disorders are the most prevalent mental disorders, accounting for over 300 million and 280 million, respectively [5]. The age-standardized prevalence of mental disorders per 100,000 people in 2019 was reported high in Australasia (17,506.6), Tropical Latin America (15,909.4), and high-income North America (15,445.8) [5]. In Sub-Saharan Africa, North Africa and Middle East have high prevalence of depressive disorders than other regions.

The community access to mental health services should be optimized to reduce the morbidity and mortality linked to mental disorders [6,7]. The integration of mental health services into primary care and the coverage of mental health services in national health insurance are just two examples of the impressive efforts undertaken by some nations to maximize this access [8]. However, a lack of community knowledge and stigmatizing attitudes toward mental illness may discourage people from getting treatment, prolong social exclusion, and create barriers to mental health access and care [9–14].

Untreated mental disorders due to the lack of access to mental health services may have a profound negative impact on individuals, families, and society at large. Including deteriorating of physical and mental health, longer recovery time, social isolation and relationship strain, impaired functioning in daily life, increased healthcare costs, loss of productivity, and increased burden on family members [15].

In Africa context, community poorly understands mental disorders, most of African societies perceive mental disorders to be associated with supernatural causes, such as demons, curses, witchcraft, and God’s punishment [9,16]. The community knowledge and attitude towards mental disorders are far away from the scientific view which may negatively affect mental health treatment [9–11]. Negative stigmatizing attitude and community bias towards mental health illness threaten and hinder the provision of mental health treatments [12].

Similarly, Tanzania faces a burden of mental disorders, with the Disability Adjusted Life Years (DALYs) of 1,721.6 per 100,000 population, and the Age-Standardized Suicide Mortality Rate of 8.15 per 100,000 population [17,18]. Despite this burden, the access to mental health treatment remains limited due to stigma and limited community knowledge around mental illness [16,17]. Low national budget expenditure on the mental health, low number of mental health professional, inadequate health facilities for treatment of mental health illness, inadequate psychiatric and forensic units, and inadequate community-based mental health services also challenge the availability and accessibility of mental health services in Tanzania [17]. Most people in Tanzania have limited knowledge and negative attitudes toward people with mental disorders [19]. The majority of community members relate the etiology of mental illness to supernatural causes, like demons, witchcraft, curses, and God’s punishment [16].

The future mental health programs that promote education, reduce stigma, enhance access, and ensure that individuals with mental disorders receive the care and support they need, can be better formulated by understanding the community’s knowledge and attitude toward mental illness. Therefore, the study aimed to assess the community knowledge and attitude towards mental illness and its associated factors in Dodoma urban central Tanzania.

## Materials and methods

### Study setting

The study was conducted at Makuru from July to September 2021, in Dodoma region which is located in the central zone of Tanzania. The region is bordered by the Chamwino district in the east and Bahi district in the west, with a total population of 3,085,625 and an average household size of 4.1 [20].

In Dodoma, Mirembe National Mental Health Hospital was the main health facility where the community access mental health services. The hospital has specialized in provision of mental health services and offers psychiatric referral services across the country.

### Study design

The study utilized a quantitative research approach with a community-based analytical cross-sectional study design to address the research objectives.

### Study population

The study population was the community members who residing in Dodoma urban.

### Inclusion criteria

The community members who residing at Makuru in Dodoma city, aged 18 years or above and consent to participate in the study were included.

### Exclusion criteria

Those who were known being seriously sick or mentally ill, in the manner that were not competent to provide informed consent were excluded.

### Sample size estimation

The sample size was calculated using the Kish and Leslie formula (1965), which is based on a single population proportion formula.

The formula states that:


$$n=\frac{Z^{2}P(1-P)}{\varepsilon^{2}}$$

Where by:

n = Estimated sample size, Z = z-score at 95% confidence level = 1.96, ε = Acceptable margin of error (5%), P = 15% which was the proportion of community attitude toward mental illness reported in a previous study.


$$n=\frac{1.96^{2}\times 0.15\left(1-0.15\right)}{0.0025}=196+\left[5\%\text{attrition rate }\left(10\right)\right]=206$$

From the estimated sample size, 5% of non-response rate was added (10 participants) to make an overall sample size of 206 participants. But, 2 participants did not complete the survey and remained with a total of 204 participants as a minimum sample size who were involved in this study.

### Sampling procedure

A multistage sampling technique was used to select participants for this study. In the first stage, a random sample of neighborhoods within the community was employed to ensure geographical representation. In the second stage, specific households within each selected neighborhood were chosen randomly. Finally, in each chosen household, a suitable individual aged 18 years or older was randomly selected to participate in the study until the minimum sample size of 204 participants was achieved.

### Data collection method

The interviewer administered questionnaire was used to gather the data from study participants. Three research assistants used to collect the data under the supervision of principal investigator. Prior the data collection, the 2 days orientation training was conducted to familiarize the research assistants with data collection tool and the rights of the participants. Each study participant was interviewed separate, and 15 minutes was required to complete fill the questionnaire.

### Data collection tool

A structured questionnaire adapted from the previous studies done by Puspitasari et al., (2020) in Indonesia, Gureje et al., (2018) in Nigeria, and Tesfaye et al., (2020) in Ethipia, was used for data collection [21–23]. The questionnaire has 3 section, the 1^st^ section comprised 8 items with multiple responses to assess participants’ social demographic information, 2^nd^ section comprised 10 items with binary responses to assess community knowledge, and the 3^rd^ section comprised 9 items with 5 five-point Likert scale to assess community attitude. The questionnaire was adapted by adding three modified questions with multiple options for assessing community awareness regarding symptoms, etiology, and treatment options. The questionnaire was initially created in English and then translated into Swahili using a rigorous translation and back-translation process to ensure accuracy and accessibility for participants.

### Validity and reliability

To ensure the content validity, a questionnaire was reviewed by a panel of 2 mental health and psychiatric experts. A questionnaire was pre-tested using 10% of the sample size, leading to minor modifications based on feedback to improve clarity and cultural relevance. Whereby, two items and one item were removed from the knowledge and attitude sections, respectively. The reliability test was conducted and the Cronbach’s alpha was above 0.7 for knowledge and attitude, which is acceptable.

### Variable description and measurements

#### Variable description.

The community attitude toward mental illness was the dependent variable, while community knowledge, and demographic variables such as age, sex, marital status, ethnicity, religion, education, and occupation were the independent variables of the study.

#### Variable measurements.

The participants’ social demographic data, such as sex, religion, marital status, ethnicity, and occupation were measured using a norminal scale, while the age groups, education level, and family monthly income were measured using the ordinal scale. The participants’ age was first transformed into 4 age group categories (15–24, 25–44, 45–64, and ≥ 65 years), family monthly income was transformed into 3 categories (<100,000Tsh, 100,000–500,000Tsh, and ≥ 500,000Tsh), and the participants’ education level was grouped to 5 categories (primary, secondary, high school, college, and informal education).

The community knowledge regarding mental illness was assessed using 10 items with binary responses (Yes = 1, No = 0). The correct response scored 1 and the incorrect response scored 0. The maximum knowledge score was (10) and the minimum was (0). The score of 6 points was the cutoff point, the participants who scored less than 6 points considered to have inadequate knowledge, and those who scored 6 points or above considered to have adequate knowledge.

The community attitude toward mental illness was assessed using 9 items with 5 point Likert scale (strongly disagree = 1, disagree = 2, neutral = 3, agree = 4, and strongly agree = 5). The minimum score was 9 points and the maximum was 45 points. The mean score was the cutoff point (29.67 ± 9.57), those who scored below the mean considered to have a negative attitude, and those who scored above the mean score considered to have a positive attitude.

### Data analysis

The data cleaning and management were done and no missing data was found. The study variables such as social demographic data, knowledge, and attitude were tested for normality and were all normally distributed.

The descriptive statistics were performed, to describe the participants’ social demographic information, knowledge, and attitude. The categorical data were presented using frequencies and percentages, while the continuous data were presented using mean and standard deviation.

In adjusting confounders, the bivariable and multivariable binary logistic models were used to establish the strength of association between dependent (attitude) and independent variables (social demographic factors and knowledge).

The final results were presented using Adjusted Odd’s Ratio (aOR), Confidence Interval (95% CI), and p-value < 0.05. The data analysis was performed using IBM-Statistical Package of Social Science (SPSS) version 27 software.

### Ethical consideration

On July 13, 2021, the Institutional Research Review Committee (IRREC) of the University of Dodoma granted ethical approval to conduct this study (Ref. No. MA. 84/261/31/155). Furthermore, permission from the city director of the Dodoma city and the ward executive officer was obtained for data collection.

Each participant was asked to sign informed consent form before participation and was free to withdraw from participation at any time. Participants’ values, integrity, and dignity were safeguarded throughout the study period. The anonymization and de-identification were used to ensure participants’ confidentiality and personal privacy. Additionally, the participants’ data were secured and protected against access from unauthorized persons.

## Results

### Social-demographic characteristics of study participants (n = 204)

A total of 204 participants, including 97 male (47.5%) and 107 female (52.5%), aged 18 years and above, residing in Makuru, were involved in this study with a total responding rate of 100% (n = 204). The average age of study participants was (33.3 ± 13.2) years while the minimum and maximum age of the study participants were 18 years and 72 years, respectively. The majority of participants 100(49%) were in the age group of 25–44 years.

Most of the study participants were Christian 140(68.6%) and in regarding to ethnicity, the majority were gogo 85(41.7%). Moreover, about half of participants were married 106(52%), and most of participants 94(46.1%) had primary education. Furthermore, regarding the occupation status, most of participants 97(47.5%) were merchants and the majority 84(41.2%) reported a family monthly income of 100,000–500,000Tsh. Refer Table 1.

**Table 1:** Social demographic characteristics of study participants (n = 204).

Demographic characteristics	Frequency(n)	Percent (%)
Age groups of participants		
15–24 years	62	30.4%
25–44 Years	100	49.0%
45–64 Years	34	16.7%
≥ 65 years	8	3.9%
Gender		
Male	97	47.5%
Female	107	52.5%
Religion of participants		
Christian	140	68.6%
Muslim	64	31.4%
Ethnicity		
Gogo	85	41.7%
Nyaturu	19	9.3%
Sukuma	19	9.3%
Others	81	39.7%
Marital status of participants		
Married	106	52%
Single	72	35.3%
Divorce	1	0.5
Widow	25	12.3%
Education level of participants		
Primary education	94	46.1%
Secondary education	59	28.9%
High education	4	2%
College and above	36	17.6%
No formal education	11	5.4%
Occupation status of participants		
Employed	32	15.7%
Peasant	29	14.2%
Merchant	97	47.5%
Housewife	15	7.4%
Student	8	3.9%
Unemployed	23	11.3%
Family monthly Income of study participants		
Less than 100,000/ = Tsh	109	53.4%
100,000–500,000/ = Tsh	84	41.2%
Above 5000,000/ = Tsh	11	5.4%

### Community knowledge regarding mental illness

The findings revealed that, majority of participants had adequate knowledge regarding mental illness 67.6% (n = 138). Most of respondents agreed that, psychological stress 83.8% (n = 171), and unstable temperament 68.1% (n = 139) were more likely to cause mental illness. Moreover, majority of community members agreed that, psychological problems and mental illness can occur at any age 83.8% (n = 171), mental illness and psychological problems can be prevented 61.8% (n = 126), anyone can become mentally ill 69.1% (n = 141), and positive attitude and good interpersonal relationship 77.9% (n = 159) help to maintain mental health. Refer Table 2.

**Table 2:** Participants’ response on knowledge regarding mental illness (n = 204).

Statements	Frequency (n)	Percent (%)
1. Mental health is a component of health?		
No	108	52.9%
Yes	96	47.1%
2. Anyone can become mentally ill?		
No	93	30.9%
Yes	141	69.1%
3. Psychological problems can occur at any age?		
No	33	16.2%
Yes	171	83.8%
4. Mental disorders and psychological problems can be prevented?		
No	78	38.2%
Yes	126	61.8%
5. Positive attitude, good interpersonal relationship and healthy lifestyle help to maintain mental health?		
No	45	22.1%
Yes	159	77.9%
6. Middle and order adult are less likely to develop mental illness?		
No	154	75.5%
Yes	50	24.5%
7. Mental illness may occur when an individual is under psychological stress or facing major life events?		
No	33	16.2%
Yes	171	83.8%
8. Individual with unstable temperament is more likely to have mental problems?		
No	65	31.9%
Yes	139	68.1%
9. Individual with family history of mental illness is at higher risk for developing psychological and mental illness?		
No	129	63.2%
Yes	75	36.8%
10. Mental health illness and mental disorders can be cured?		
No	104	51%
Yes	100	49%

**Fig 1:**
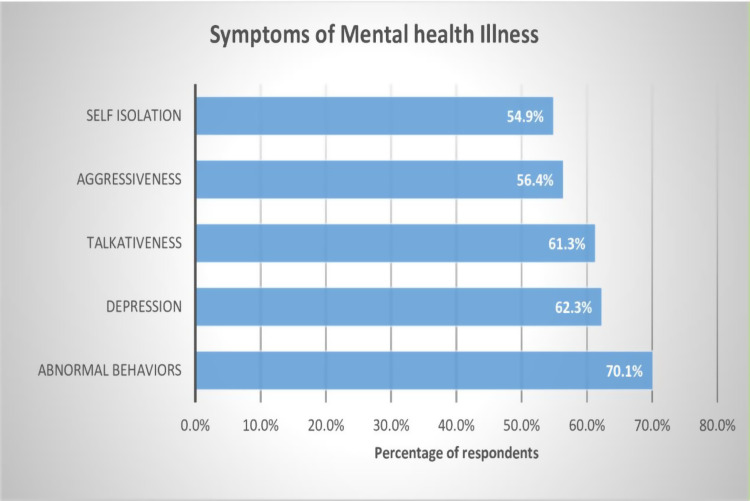
Community awareness regarding symptoms of mental illness.

On regarding to symptoms of mental illness, more than half of study participants have awareness about symptoms of mental illness such as aggressiveness, Self-isolation, abnormal behaviors, talkativeness and depression. Abnormal behaviors was identified by most of study respondents (70.1%) as a symptom of mental illness. Refer Fig 1.

**Fig 2:**
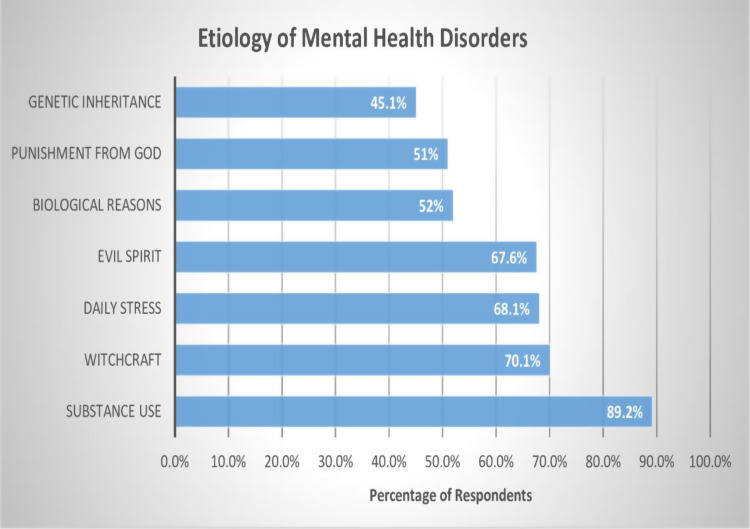
Community awareness regarding etiology of mental illness.

On regarding to etiology of mental illness, majority of study respondents reported mental illness to be attributed by witchcraft 70.1% (n = 143), Evil spirit 67.6% (138), and God’s punishment (51.5%). However, most of respondents reported substance use as etiology of mental illness 89.2% (n = 182). Refer Fig 2.

**Fig 3:**
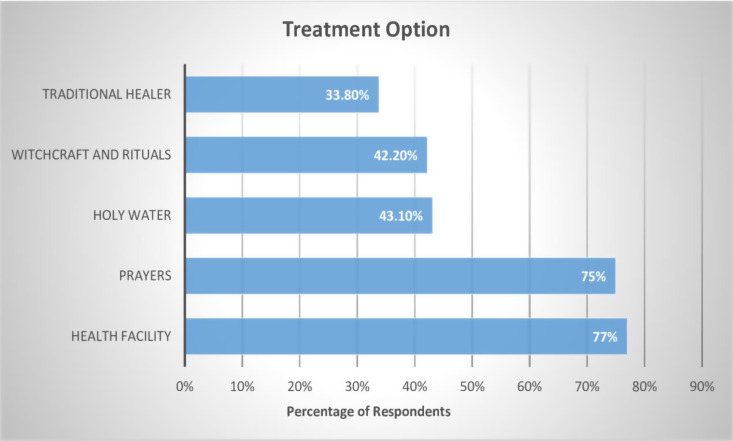
Community awareness regarding treatment option of mental illness.

On regarding to treatment option, health facilities 77% (n = 157) and prayers 75% (n = 153) were the most reported preferred treatment option for mental illness. However, the minority preferred witchcraft and rituals 42.2% (n = 86), traditional healer 33.8% (n = 69), and 43.1% (n = 88) holy water. Refer Fig 3.

### Community attitude toward mental illness

Most of the participants have positive attitude toward mental illness 113(55.4%) compared to respondents who had negative attitude 91(44.6%). The social stigmatizing attitudes towards people with mental illness include unwilling to share a room 106(64.7%), unwilling to marry 164(80.4%), unwilling to make friendship 83(40.7%), unwilling to live 66(32.4%), unwilling to work 98(48%), and unwilling to visit a mentally ill person 125(61.3%). Refer Table 3.

**Table 3:** Participants’ responses regarding attitude toward mental illness (n = 204).

Statements	SD	D	N	A	SA
1. Can you live with a person who has mental illness	50	5	11	14	124
2. Can you make friendship with a person who has a mental disorder	61	12	10	14	107
3. Can you work with a person who has a mental disorder	73	9	16	17	89
4. Can you accept a person who has a mental disorder to visit you	45	0	2	10	147
5. Can you visit a mental ill person	99	9	17	0	79
6. People with mental illness supposed to have kids	69	6	7	5	117
7. Can you share a room with a person who has mental disorder	95	0	11	5	93
8. Can you have greeting with mentally ill person	42	2	0	10	150
9. Can you marry someone with mental illness	154	2	8	2	38

SD = Strongly disagree, D = Disagree, N = Neutral, A = Agree, SA = Strongly agree

### Social-demographic factors associated with community knowledge regarding mental illness

After adjusting confounders in multivariable binary logistic regression model, the results revealed that, sex [aOR = 5.85: 95% CI; 1.77–19.34: p = 0.004], religion [aOR = 0.36: 95% CI; 0.14–0.93: p = 0.034], marital status [aOR = 0.16: 95% CI; 0.03–0.9: p = 0.039], and occupation [aOR = 18.69: 95% CI; 2.10–166.79: p = 0.009] were identified social demographic factors associated with the community knowledge regarding mental illness.

From Table 4; the study participants who were male sex were 5.85 times more likely to have inadequate knowledge than female [aOR = 5.85: 95% CI; 1.77–19.34: p = 0.004]. Moreover, participants who were Christian were less likely to have inadequate knowledge compared to Muslim participants [aOR = 0.36: 95% CI; 0.14–0.93: p = 0.034]. Furthermore, participants who were married were less likely to have inadequate knowledge than those were widow [aOR = 0.16: 95% CI; 0.03–0.9: p = 0.039]. Also, participants who were housewife were 18.69 times more likely to have inadequate knowledge than those who were unemployed [aOR = 18.69: 95% CI; 2.10–166.79: p = 0.009].

**Table 4:** Bivariable and multivariable binary logistic regression analysis showing social demographic factors associated with community knowledge regarding mental illness (n = 204).

Variables	cOR	95% CI		*P*-value	aOR	95% CI		*P*-value
		Lower	Upper			Lower	Upper	
Age groups of participants								
15–24 years	0.084	0.010	0.025	0.025	0.440	0.023	8.498	0.587
25–44 Years	0.040	0.005	0.003	0.003	0.073	0.004	1.406	0.083
45–64 Years	0.100	0.011	0.041	0.041	0.168	0.009	3.124	0.231
≥ 65 years	1				1			
Sex								
Male	1.156	0.643	0.628	0.628	5.854	1.770	19.366	**0.004**
Female	1				1			
Religion								
Christian	0.585	0.315	0.089	0.089	0.356	0.137	0.925	**0.034**
Muslim	1				1			
Ethnicity								
Others	0.766	0.396	1.480	0.427	0.615	0.238	1.588	0.315
Sukuma	0.515	0.157	1.694	0.275	0.468	0.084	2.603	0.386
Nyaturu	2.146	0.785	5.867	0.137	4.537	0.874	23.550	0.072
Gogo	1				1			
Marital status								
Married	0.270	0.109	0.005	0.005	0.160	0.028	0.913	**0.039**
Single	0.587	0.235	0.255	0.255	0.688	0.103	4.588	0.700
Divorce	9.972E-7	9.972E-7			2.671E-7	2.671E-7	2.671E-7	
Widow	1				1			
Education level								
Primary education	0.745	0.212	0.646	0.646	2.823	0.200	39.853	0.442
Secondary education	0.823	0.225	0.768	0.768	1.824	0.106	31.267	0.678
High education	0.400	0.031	0.482	0.482	2.722	0.053	140.080	0.619
College and above	2.287E-10	2.287E-10			7.782E-13	0.000	.	0.989
No formal education	1				1			
Occupation status								
Employed	0.480	0.160	0.190	0.190	0.836	0.145	4.833	0.841
Peasant	0.745	0.248	0.599	0.599	2.190	0.322	14.892	0.423
Merchant	0.253	0.098	0.005	0.005	0.916	0.190	4.416	0.913
Housewife	1.375	0.368	0.636	0.636	18.693	2.095	166.789	**0.009**
Student	4.749E-10	4.749E-10			45151.831	0.000	.	0.998
Unemployed	1				1			
Family monthly Income								
Less than 100,000Tsh	0.669	0.192	0.528	0.528	4.782E-08	0.000	.	0.991
100,000Tsh–500,000Tsh	0.426	0.118	0.192	0.192	9.639E-08	0.000	.	0.991
Above 5000,000Tsh	1				1			
Attitude status								
Negative	1.985	1.095	3.596	0.024	2.005	0.775	5.189	0.152
Positive	1				1			

### Social-demographic factors associated with community attitude toward mental illness

After adjusting confounders in multivariable binary logistic regression model, the results revealed that, age [aOR = 9.94: 95% CI; 1.04–94.97: p = 0.046], ethnicity [aOR = 4.45: 95% CI; 1.20–16.53: p = 0.026], and occupation [aOR = 0.19: 95% CI; 0.04–0.85: p = 0.029] were the social demographic factors associated with community attitude toward mental illness.

From Table 5; the study participants who were aged 25–44 years were 9.94 times more likely to have negative attitude than those who were aged 65 years and above [aOR = 9.94: 95% CI; 1.04–94.97: p = 0.046]. Moreover, the participants who were nyaturu were 4.45 times more likely to have negative attitude than those who were gogo ethnicity [aOR = 4.45: 95% CI; 1.20–16.53: p = 0.026]. Also, participants who were employed were less likely to have negative attitude compared to unemployed study participants [aOR = 0.19: 95% CI; 0.04–0.85: p = 0.029].

**Table 5:** Bivariable and multivariable binary logistic regression analysis showing social demographic factors associated with community attitude toward mental illness (n = 204).

Variables	cOR	95% CI		*P*-value	aOR	95% CI		*P*-value
		Lower	Upper			Lower	Upper	
Age groups of participants								
15–24 years	0.442	0.100	1.955	0.282	0.945	0.106	8.394	0.960
25–44 Years	1.326	0.314	5.602	0.702	9.936	1.040	94.965	0.046
45–64 Years	0.478	0.100	2.278	0.354	2.255	0.246	20.659	0.472
≥ 65 years	1				1			
Sex								
Male	0.522	0.835	0.480	1.452	0.894	0.378	2.110	0.797
Female	1				1			
Religion								
Christian	0.728	0.402	1.319	0.296	0.897	0.381	2.109	0.803
Muslim	1				1			
Ethnicity								
Others	1.326	0.716	2.453	0.369	1.502	0.643	3.511	0.348
Sukuma	0.536	0.177	1.625	0.270	1.031	0.277	3.832	0.964
Nyaturu	4.200	1.385	12.738	0.011	4.452	1.199	16.533	0.026
Gogo	1				1			
Marital status								
Married	0.356	1.528	0.620	3.765	1.998	0.479	8.331	0.342
Single	0.394	1.504	0.588	3.847	3.377	0.623	18.294	0.158
Divorce		7.989E-9	7.989E-9	7.989E-9	2.109E-8	2.109E-8	2.109E-8	
Widow	1				1			
Education level								
Primary education	0.703	0.200	2.463	0.581	0.271	0.045	1.644	0.156
Secondary education	0.923	0.253	3.359	0.903	0.533	0.071	4.019	0.542
High education	5.054E-8	5.054E-8	5.054E-8		4.911E-8	0.000	.	0.998
College and above	0.201	0.047	0.854	0.030	0.106	0.011	1.066	0.057
No formal education	1				1			
Occupation status								
Employed	1.064	0.356	3.181	0.911	0.191	0.043	0.847	0.029
Peasant	1.452	0.479	4.405	0.510	1.169	0.249	5.484	0.843
Merchant	1.139	0.450	2.884	0.784	0.744	0.195	2.845	0.666
Housewife	10.111	1.832	55.795	0.008	8.412	0.901	78.499	0.062
Student	0.222	0.023	2.122	0.191	0.364	0.020	6.569	0.493
Unemployed	1				1			
Family monthly Income								
Less than 100,000Tsh	2.716	0.684	10.785	0.156	0.521	0.059	4.589	0.557
100,000Tsh-500,000Tsh	1.725	0.427	6.978	0.444	0.203	0.021	1.969	0.169
Above 5000,000Tsh	1				1			
Knowledge status								
Inadequate	1.985	1.095	3.596	0.024	1.995	0.825	4.824	0.125
Adequate	1				1			

## Discussion

The findings from this study revealed that, majority of study respondents have adequate knowledge and positive attitude toward mental illness. The level of stigmatizing attitudes toward people with mental illness was high among respondents. Also, perceived etiology of mental illness and treatment options were far away from scientific view. These findings were in line with previous studies conducted in Northern Tanzania [16], South-West Nigeria [11], Western Ethiopia [9], and Bungoma Kenya [24].

High results on knowledge evaluation revealed that the majority of study participants had a decent understanding of mental illness. This study had a lower percentage of respondents with inadequate knowledge compared to studies done in Enugu-Nigeria [25], and Bungoma-Kenya [24]. The variation in levels of knowledge may be explained by variations in study population, setting, and demographics. Community awareness is essential in influencing attitudes, perceptions, and behavior related to seeking out health.

Negative attitudes can be influenced by a lack of awareness regarding mental health illnesses [26]. Enhancing community understanding of mental illness is a key aspect for enhancing community health-seeking behavior. Public mental health campaigns, community-based mental health organizations, media education, and community mental health rehabilitation facilities are just a few of the strategies that can help the community to become more empowered and informed.

Although some survey participants displayed positive attitudes towards mental illness, a sizable portion nevertheless held unfavorable ideas and stigmatizing attitudes. The results of numerous studies conducted in Nigeria [11] and Yoruba South Western Nigeria which also revealed social stigmatization of people with mental illness, were consistent with this study [23].

Unfavorable community attitude may led to subpar community health seeking behavior, insufficient mental health promotion, insufficient treatment and rehabilitation services, decreased accessibility and use of mental health services, and a general decline in community mental health. Using tactics like offering mental health education, forming community-based mental health organizations, and launching public mental health campaigns, with the focus of etiology and treatment option of mental illness can improve the social climate, reduce stigma, and change the community attitude.

The study discovered that positive community attitude toward mental diseases was significantly influenced by ethnicity. This implies that cultural influences, such as ethnic background, can affect knowledge and understanding of mental health illnesses [27]. Communities that place more of a focus on mental health education and awareness, or those with better access to mental health resources and information, may help their residents become more knowledgeable about mental diseases.

Aspects of particular ethnic groups’ cultural values, beliefs, and traditions may also have an impact on how people see and understand mental health [27]. It may be possible to establish tailored interventions and educational initiatives to improve mental health literacy across various groups by furthering our understanding of the precise mechanisms by which ethnicity affects mental health knowledge.

The study found that gender substantially impacted knowledge of mental diseases, with more males displaying higher levels of knowledge than females. This data raises the possibility of gender-related variations in the knowledge and comprehension of mental health conditions which was also reported in previous studies [26,28,29]. This disparity may have different access to mental health information, different help-seeking behaviors, or different cultural expectations and roles placed on each gender in relation to mental health, among other factors.

To better understand how gender influences mental health knowledge and to develop strategies that effectively increase mental health literacy among males and females, it is crucial to look into these issues in more detail. Such initiatives can aid in lowering the stigma attached to mental diseases and promoting a more aware and understanding society.

According to the study’s findings, occupation status was a significant factor that was linked to a greater understanding of mental diseases, with more housekeepers displaying higher levels of knowledge than peasants, merchants, students, and those without a job. This shows that people in particular professions might be exposed to more information and resources on mental health [26]. Housekeepers may have more opportunity to learn about mental health illnesses since they are more likely to live in environments where they interact with various people or have access to educational materials.

Contrarily, people in other professions or those without jobs might have limited access to these resources, leading to lesser knowledge. In order to increase awareness of mental health issues and advance general well-being, it can be helpful to understand these occupational disparities to direct targeted interventions and awareness campaigns suited to particular occupational groups.

Some of studies have shown a significant association between community knowledge and attitude toward mental illness. The poor community understanding of mental illness has greater influence on negative community attitude [26]. However, the finding from this study does not report the significant association between community knowledge and attitude. The variation in these findings might be caused by their variations in the study population, setting, and demographics.

This study emphasize the integration of mental health services into primary healthcare settings. Together with physical health, mental health should be addressed, and medical personnel should receive training on how to identify mental health issues and provide non-stigmatizing support or referrals. By establishing mental health care as a component of general health, policies may concentrate on increasing accessibility and reducing stigma attitudes.

Additionally, local healthcare systems should put in place community-based mental health initiatives that emphasize providing help in table settings and lowering stigma. People who might shun conventional psychiatric services because mental illness is stigmatized could be reached by such initiatives.

## Conclusion

The study revealed that, more than half of respondents displayed a common stigmatizing attitude of not willing to share a room, marry, or visit a mentally ill person, despite the participants’ adequate knowledge and favorable attitude. Using strategies like provision mental health education, forming community-based mental health organizations, and launching public mental health campaigns will help to improve community mental health awareness and reduce negative attitude.

### Limitations

This study solely included urban residents and employed a single research approach (quantitative) with a limited sample size of 204 participants. These could introduce biases into the study and restrict how far the findings can be applied. Consequently, care should be taken when interpreting the study’s findings. Future research should concentrate on a mixed research approach, a large sample size, and both rural and urban population.

## Supporting information

S1 Data Data template of study participants.
(SAV)
